# Anatomical patterns of cleft lip and palate deformities among neonates in Mekelle, Tigray, Ethiopia; implication of environmental impact

**DOI:** 10.1186/s12887-019-1624-2

**Published:** 2019-07-24

**Authors:** Konjit K. Bekele, Peter E. Ekanem, Berhanu Meberate

**Affiliations:** Department of Anatomy, College of Health Sciences, Mekelle University, P.O. Box 1674, Mekelle, Ethiopia

**Keywords:** Anatomical patterns, Cleft lips, Cleft palate, Neonates

## Abstract

**Background:**

Cleft lip and palate deformities are considered one of the most common birth defects of the head and neck that pose significant medical, psychosocial and financial burdens on the affected individuals and families, especially in low income communities. The etiology and pathogenesis of cleft lip and palate is complex and is known to involve genetic and/or environmental factors.

**Objective:**

To assess the patterns of anatomical cleft lip and palate deformities among neonates in Mekelle and Ayder Comprehensive Specialized hospitals, Tigray, Northern Ethiopia.

**Methods:**

A hospital-based retrospective study was conducted from May 2017 to June 2017 at Mekelle and Ayder Comprehensive Specialized hospitals, both in Mekelle city. Data was collected from all medical charts of neonates registered from 2011 to 2016 and analyzed using SPSS version 21.0 and OpenEpi software. Results were presented using tables and graphs; Chi-square test was used to look for an association between variables, odds ratio to determine the strength of association of selected variables using multinomial logistic regression model, while Fisher Exact (Clopper-Pearson) was used to compare yearly prevalence.

**Results:**

Of 37,152 neonatal charts analyzed, 119 (0.32%) cases were identified as having cleft deformities. 38.7, 17.6, and 43.7% of this figure had cleft lips, cleft palates and both cleft lip and palate respectively. 46 (38.7%) neonates had lateral patterns of cleft lip deformities with 56.5% located unilaterally on the right and 43.5% unilaterally on the left. Of 52 (43.7%) neonates with cleft lip and palate deformities, 40.4% were located bilaterally while 38.5 and 21.2% were located unilaterally on the left and right, respectively. Associated malformations were: cardiac (3.4%), central nervous system (1.7%) and limb deformities (5.9%). The overall prevalence of cleft deformities was found to be 3.11 per 1000 live births.

**Conclusion:**

The study showed a higher prevalence of cleft deformities than that reported in Addis Ababa and some other African countries. A higher occurrence of left unilateral pattern of cleft lip and palate was observed whereas a higher right unilateral pattern of cleft lip was identified. The higher prevalence of cleft lip and palate recorded in this region of Ethiopia may reflect an environmental impact.

## Background

Birth defects are one of the leading causes of infant mortality in the world, contributing to more than 3 million deaths among children aged < 5 years [1]. According to the World Health Organization (WHO) in 2010, an estimated 270,000 neonatal deaths globally were attributable to congenital anomalies. Congenital malformations of the head and neck with cleft lip and palate anomalies (CLP) are considered the most common forms of birth defects. CLP are derived embryologically, from defects in the primary fusion of the craniofacial processes that form the primary and secondary palate between the 5th and 12th weeks of development. Based on the anatomy of the incisive foramen as a landmark, CLP are classified into: pre-incisive foramen or clefts lip (CL), post-incisive foramen or cleft palate (CP), trans-incisive foramen or cleft lip and palate (CL/P) [2]. CLP are also designated clinically by their location and descriptive terms, such as unilateral, bilateral, or midline and complete, incomplete, or sub-mucus. [3] Clefts occur in 2 groups: syndromic or isolated. Syndromic clefts are typically accompanied by abnormalities in other developmental fields or organ systems e.g., limbs, central nervous system (CNS), cardiovascular system (CVS), etc.

Structurally, CL results from a lack of fusion of maxillary and nasomedial processes. The mechanism frequently underlying CL is hypoplasia of the maxillary process that prevents contact between the maxillary and nasomedial processes. It may also result from deficiency in the merging of several mesenchymal masses and proliferations to smoothen out the overlying epithelium. Cleft palate, on the other hand results from incomplete or absence of fusion of the palatal shelves [4].

Etiology and pathogenesis of CLP are poorly understood and compass both genetic and environmental factors. Genetic factors associated with CLP have been identified in several research works and include: TGF-β3 (Transforming growth factor beta 3), MSX1 (Msh homeobox1), IRF-6 (Interferon regulatory factor- 6), FGFs (Fibroblast growth factor), PVRL1 (Poliovirus receptor related-1), FOXE1 (Forkhead box E1), JAG2 (Jagged 2) and TBX22 (T-box22) [2]. Environmental factors such as mother’s diet, vitamin supplementation, and use of alcohol, smoking, and anticonvulsant drugs have been implicated in the development and high incidence of CLP. CLP generally have an aesthetic significance and impact which pose significant medical, psychosocial and financial challenges on both the patients and caregivers [1, 3]. The complications associated with CLP include feeding difficulties which appear after birth due to anatomical defective structures impairing suckling and swallowing. Others are: scarring, dental problems, speech and hearing impairments, deficient maxillofacial growth and aesthetic challenges, which may attract bullying among peers and segregation. These factors consequently lead to low self-esteem and low quality of life indices [5].

Global prevalence of cleft lip and palate deformities ranges from 2.7 to 9.92 cases per 1,000 live births [6] with variability by country, race, geographical location, gender and ethnic group. The lowest reported incidence is among African–American populations (approximately 0.5 per 1,000) [7, 8] while a higher incidence exists among native Americans (approximately 3.5 per 1,000) [9–12].

Although a number of research studies have been carried out to determine the prevalence of CLP in several African countries including Ethiopia (Addis Ababa), none has been conducted in Mekelle (a city in Northern Ethiopia), where a large number of orofacial defects has been seen. This has prompted this study aimed at assessing the patterns and prevalence of CLP among neonates born in or attending ACSH and MH, Mekelle, for the purpose of devising correct surgical interventions and prevention of these defects.

## Methods

The present study was conducted in two main government hospitals: MH and ACSH in Tigray regional state of Northern Ethiopia from May 2017 to June 2017. ACSH is the second largest hospital in Ethiopia, serving a catchment population of over 8 million people from Tigray, Afar and the Southeastern part of Amhara states. MH serves over 2 million people mainly from Tigray state. A retrospective study with purposive sampling technique was employed. The study population included all neonates with cleft lip and palate deformities registered from 2011 to 2016. All affected neonates with complete chart documentation were included while those with missing pertinent data were excluded.

An adapted structured checklist was used to extract data from delivery registration books and medical records. Variables studied were cleft deformities and associated anomalies like congenital heart defects, central nervous system (CNS) and extremity malformations as they relate to patients’ sex. 37,152 birth and attendance records from 2011 to 2016 were retrieved. The data collected were analyzed using SPSS™ software (version 21.0) and Open Epi™. For descriptive purposes, results were presented as frequency and percentages in tables and graphs. Fisher Exact (Clopper-Pearson) compared the yearly prevalence, Chi-square tested association between variables while Odds ratio was used to determine the strength of association of selected variables using multinomial logistic regression model. *P* < 0.05 was considered statistically significant.

Ethical clearance was obtained from the Ethical Review Board of Mekelle University, College of Health Sciences and an official letter of support was sent to ACSH and MH. Confidentiality was maintained throughout the study process.

## Results

In total, there were 37,152 registered neonates during 2011–2016 at ACSH and MH, and 119 (0.32%) of neonates were identified with orofacial clefting (CL, CP and CL/P). The overall prevalence from these two facilities from 2011 to 2016 was 3.11 per 1000 live births.

## Discussion

**Fig. 1:**
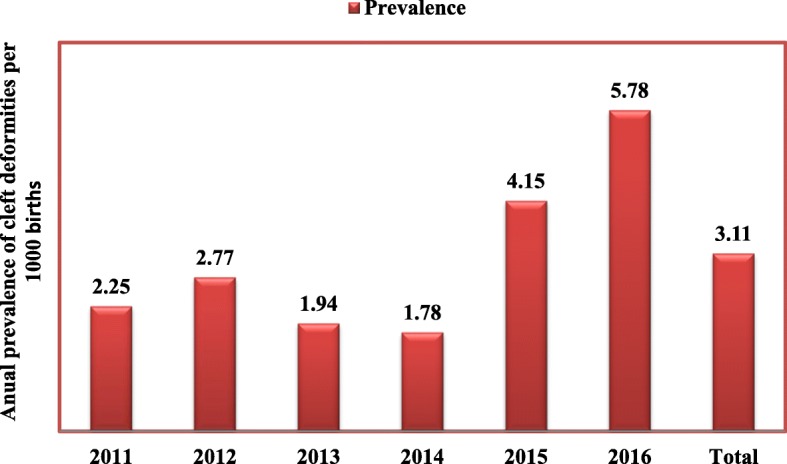
The prevalence of CLP among the neonates delivered in ACSH and MH in 2011–2016. *(The prevalence was: in 2011, 0.23 [CI: (0.06–0.72)]; in 2012, 0.28 [CI: (0.06–0.72)]; 2013, 0.19 [CI: (0.02–0.56)]; 2014, 0.18 [CI: (0.02–0.56)]; 2015, 0.42 [CI: (0.16–1.02)]; 2016, 0.58 [CI: (0.22–1.16))*

The prevalence of orofacial clefts differs among different populations. In the present study, distribution of oral clefts was seen in 0.32% of 37,152 registered neonates between 2011 and 2016 at ACSH and MH (Fig. 1). The overall prevalence was 3.11 per 1,000 live births indicating a higher figure compared to those obtained in Caucasian populations (approximately 1 per 1000 births) and Asian populations (approximately 1.7 per 1000 births) [10, 13]. Nigerian literature reviewed revealed that the prevalence of cleft anomalies was approximately 0.37/1,000 live births, a Ugandan study reported 1.45/1,000 live births, Tunisia, 1.40/1,000 live births and South Africa, 0.3/1000 live births [14–17]. In an earlier study conducted at Addis Ababa Ethio-Swedish children’s hospital, 8% of children under study were treated for cleft deformities [18] and prevalence of CLP was approximately 1.49/1,000 live births [5]. Contributing discrepancy between the findings in Addis Ababa as a cosmopolitan city and Mekelle may be due to heterogeneous population in Addis Ababa, differences in time period, socio-economic status, sample size and study design. The lower regional prevalence of CLP in Addis Ababa may also be associated with improved living conditions hence reducing the risk of developing CLP. The data in our study indicated a surge in the prevalence recorded in 2015 and 2016 compared with previous years (Fig. 1). Analysis of this prevalence did not reveal any significant difference from the previous years. We conclude that the surge may have resulted from increased use and attendance at both facilities occasioned by societal education and improved healthcare services introduced at these hospitals to guarantee safe delivery and patient care.

**Fig. 2:**
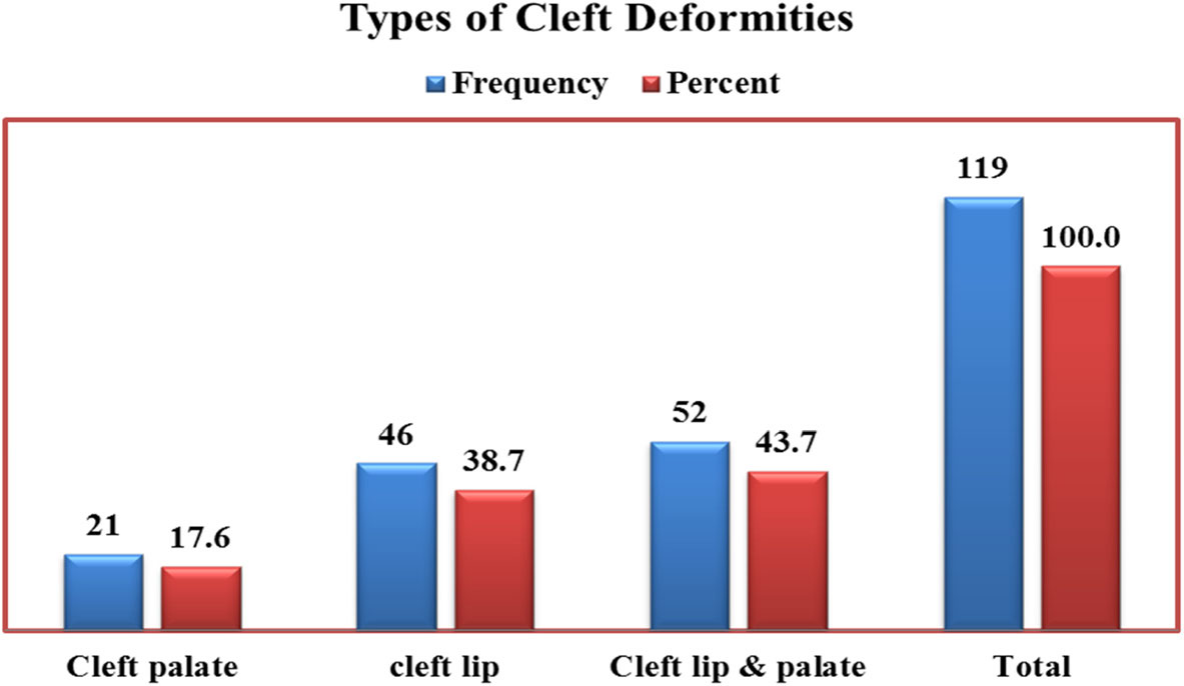
Types of cleft among neonates in ACSH and MH in 2011–2016

We found that of the 119 cases of clefting, 43.7% had CL/P, 38.7% had CL and 17.6% CP deformities, signifying a higher frequency of CL/P deformities than CL and CP (Fig. 2). In Addis Ababa, a higher incidence of CL/P than CL and CP was recorded [5]. Other studies conducted in South Indian population showed similar results, indicating CL/P being the most frequently occurring cleft anomaly, thus corroborating our result [19–21]. A contrasting view has been promulgated by Khajanchi et al. who asserted in their work that occurrence of CL was the most common cleft abnormality followed by CL/P [22]. Environmental factors have been associated with increased risk of CL/P including: consanguinity, smoking, alcohol ingestion, use of anticonvulsants during pregnancy as well as insufficient folic acid intake in the pregestational period and first trimester of pregnancy [23]. Mekelle city is fast evolving as the commercial hub of the northern part of Ethiopia with high rate of smoking, alcohol use and other forms of social indulgences. These factors may precipitate a high rate of CL/P found in Mekelle as in other commercial capitals of the world.

In this study, cleft deformities were found to be more common in males (52.9%) than females (47.1%) Table 1. Similar findings were described in Addis Ababa and among four provinces in West and Northwest Iran [5, 24]. The male: female ratio for CL, CP and CL/P in our study was 1:1.19, 1:0.5 and 1:0.86, respectively, revealing a higher occurrence of CP and CL/P among males than females while the female to male ratio in CL was higher. In contrast, more females were reported to have CP than males in Addis Ababa [5, 18] and Lahore, Pakistan [25]. A higher prevalence of milder CP was found in males as opposed to more severe forms in which females were more prevalent in a Japanese study [26]. The higher prevalence of CP in males found in our study may also be associated with occurrence of milder forms of the anomaly though this was not evaluated.

**Table 1:** Frequency of cleft deformities among neonates in ACSH and MH based on sex and cleft deformity types

Characteristics (*N* = 119)	Category	Sex of live births		Total
		Male	Female	
Type of Cleft lip	Cleft lip	21(17.6%)	25(21%)	46(38.7%)
	Cleft palate	14(11.8%)	7(5.9%)	21(17.6%)
	Cleft lip and palate	28(23.5%)	24 (20.2%)	52 (43.7%)
Total		63 (52.9%.)	56 (47.1%)	119 (100%)

**Fig. 3:**
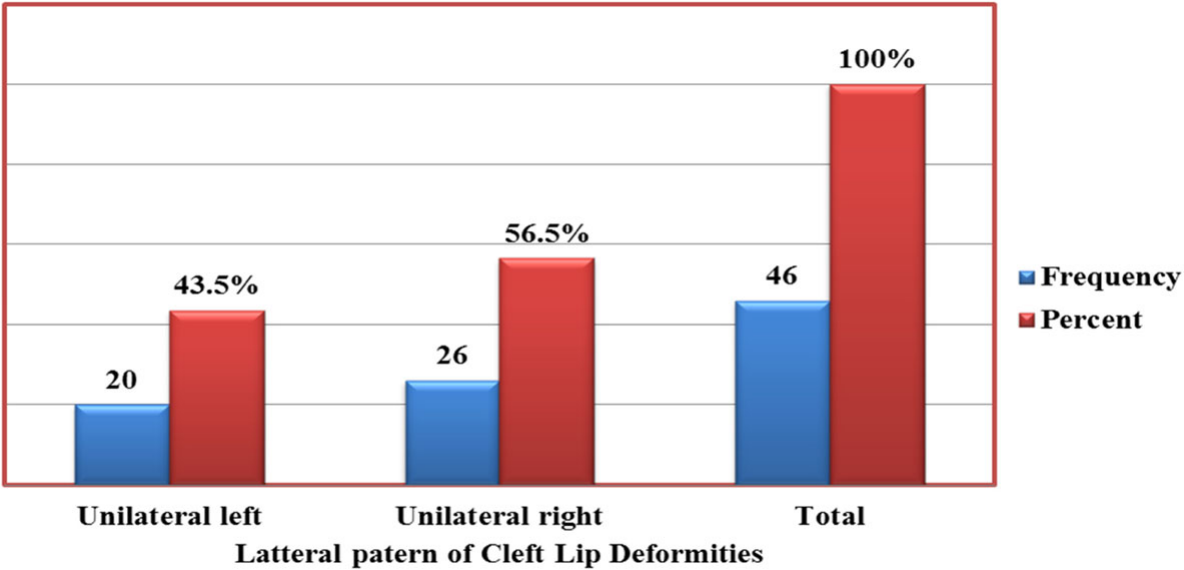
Lateral patterns of cleft lip deformities

56.5% of cleft lip deformities in this study were found unilaterally on the right side while 43.5% were located on the left (Fig. 3). Our result here is divergent to widely reported higher left sided unilateral clefts than right-sided CLs [27–29]. Majority (40.4%) of CL/P were located bilaterally. Unilateral patterns had a left sided dominance (38.5%) while right sided was 21.2% (Table 2). This finding is validated by the results obtained in Addis Ababa where 40.5% of CL/P were located bilaterally, 33.3% unilaterally on the left side and 27.8% unilaterally on the right. Other authors have, however, reported higher occurrences of left unilateral CL/P than right sided and bilaterally [5, 30, 31].

**Table 2:** Lateral patterns of CLP deformities among neonates in MH and ACSH, 2011–2016

	Category	Number (%)
Lateral pattern of cleft lip and palate deformities	Unilateral Right	11 (21.15%)
	Unilateral Left	20 (38.46%)
	Bilateral	21 (40.38%)
Total		52 (100%)

11.0% of neonates with CLP had associated congenital anomalies (Table 3). Limb malformations were most commonly seen at 5.9% followed by congenital heart defects (3.4%) and CNS malformations (1.7%). Associated malformations of CLP were recorded in other countries like Jordan [32]. In Addis Ababa, 14.1% of CLP patients had associated congenital anomalies with 7.8, 4.8 and 1.6% limb, CNS and cardiac malformations respectively [5]. In contrast, an Iranian study had 13% of CLP neonates associated with other congenital anomalies of the head, face and heart [33]. The higher incidence of limb malformations may be due to their association with neural tube defects, whose prevalence has been found to be as high as 26 times the normal non-folic acid preventable rate in Tigray region, Northern Ethiopia [34].

**Table 3:** Associated congenital malformations among neonates with cleft lip and palate deformities based on sex at MH and ACSH, 2011–2016

Characteristics	Category	Sex of neonate		Total
		Male	Female	
Live births malformation	Congenital heart defect (*N* = 4)	1 (25%)	3 (75%)	4 (100%)
	Central nervous malformation (*N* = 2)	2 (100%)	0 (0%)	2 (100%)
	Extremity malformation (*N* = 7)	5 (71.4%)	2 (28.6%)	7 (100%)
Total		8	5	13

In this study we tried to associate patients’ sex with the occurrence of cleft lip and palate deformities. Our analysis did not show any statistically significant association as shown in Table 4**.** Martelli et al. found in their work that there were significant differences in the distribution of non-syndromic cleft lip and/or palate between males and females; CP were frequent in females while CLP predominated in males [35]. Association between cleft lip severity and sex was found in the study conducted by Carroll and Mossey, with females having a significantly greater proportion of CL and CL/P in males [36].

**Table 4:** Association between sex and CLP

Characteristics (*n* = 119)	Categories	Sex of neonates		COR (95% CI)	*P*-Value
		Male	Female		
Types of cleft deformities	Cleft lip	21	25	0.72 (0.32–1.59)	0.42
	Cleft palate	14	7	1.71 (0.59–4.94)	0.32
	Cleft lip & palate	28	24	1.00^+^	1.00^+^

When sex was associated with cleft lip and palate deformities using multinomial logistic regression analysis, it was not found to be significant (*p* > 0.05)

1.00^+^ = Reference

Environmental factors in Tigray region of Ethiopia may have a strong correlation with the development of clefting. This is because folic acid, which is found in green vegetables and fruits, is one of the vital nutrients that have been found deficient among Ethiopian women [37]. Catechins, found in tea consumed generally by the people of this region several times a day, also inhibit intestinal folate absorption. When consumed by women in the periconceptional period as is commonly found in this region it may predispose them further to folate deficiency and CLP [38]. The influence of environmental factors on the incidence of CLP has been elucidated by experiments conducted in mice, which showed that the likelihood of occurrence of CP after exposure to a dose of cortisone strongly correlated to the genetic background of the mice. In humans, mutations of MSX1 gene are strongly associated with non-syndromic cleft palate [4]. Biological activities linked to orofacial pathology in certain genes but without direct involvement have been identified in folic acid receptor 1 (FOLR1). Susan et al. demonstrated through their research that perturbations of the genes in the folate pathway might contribute to the development of non-syndromic cleft lip with or without cleft palate (NSCLP) [39]. It has been shown that folate supplementation to women during the periconceptional period reduced the incidence of CL/P substantially as compared to that in the non-supplemented controls [40].

Therefore, these environmental factors highlighted may have a significant role in the high prevalence of CLP in Mekelle and Tigray region of Ethiopia.

## Conclusions

The overall prevalence of cleft deformities observed in ACSH and MH, reflective of their catchment populations, was relatively higher than what other studies have reported. A higher occurrence of clefting was observed in males than females. The influence of environmental factors and nutrition may have contributed to the high incidence.

Periconceptional administration of folate in this region as a policy may help alleviate the problems associated with orofacial development significantly.

The scope of the present study was limited to the two health facilities in Mekelle, Tigray region of Ethiopia. Acquisition of pertinent maternal history, which may have been useful to broaden the study variables and strengths of association, was also limited by insufficient data. These may explain some of the variations in this work when compared with other studies.

## Data Availability

Datasets generated and analyzed in this work can be found both in Mekelle and Ayder Comprehensive Referral Hospitals. Restrictions apply to the availability of the data for confidentiality purposes, and are thus not publically available. Data are however available upon reasonable request with permission from the respective hospitals.

## Abbreviations

ACSH: Ayder Comprehensive Specialized Referral Hospital
CL: Cleft lip
CL/P: Cleft lip and palate
CLP: Cleft lip and palate deformities
CNS: Central Nervous System
CP: Cleft palate
MH: Mekelle Hospital
NSCLP: Non syndromic cleft lip and palate
